# The mediating effect of food choice upon associations between adolescent health-related quality of life and physical activity, social media use and abstinence from alcohol

**DOI:** 10.1186/s12955-023-02129-7

**Published:** 2023-05-18

**Authors:** Jenny Davison, Brendan Bunting, Barbara Stewart-Knox

**Affiliations:** 1 Psychology Research Institute, Ulster University, Cromore Road, Coleraine, BT52 1SA Northern Ireland, UK; 2 Division of Psychology, University of Bradford, Bradford, BD7 1DP West Yorkshire UK

**Keywords:** Health-related quality of life, Kidscreen52, Food choice, Physical activity, Social media, Alcohol, Adolescents

## Abstract

**Background:**

Understanding how health-related quality of life (HRQoL) is related to lifestyle factors during adolescence is crucial to effective health promotion. The aim of this analysis was to identify associations between HRQoL and lifestyle and to determine the degree to which they are mediated by food choices in adolescents.

**Methods:**

The Wellbeing in Schools (NI) survey (N = 1609; 13–14 years) assessed HRQoL using the Kidscreen52. Food choice was assessed by Food Frequency Questionnaire (FFQ) and physical activity was assessed using the Physical Activity Questionnaire for Adolescents (PAQ-A). Social media and alcohol abstinence were self-reported.

**Results:**

Path analysis indicated that fruit and vegetable intake was associated with higher HRQoL on dimensions of moods and emotions, parent relations and home life, financial resources, and social support and peers. Bread and diary intake was related to higher physical wellbeing. Protein was associated with higher psychological wellbeing, moods and emotions, self-perception, parent relations and home life, financial resources, and lower social support and peers. Junk food was related to lower moods and emotions. Males had higher psychological wellbeing, moods and emotions, parental relations and home life. Females had higher self-perception, autonomy, and social support and peers. Greater physical activity explained higher HRQoL on all dimensions. Less social media was associated with higher psychological wellbeing, moods and emotions, self-perception, parent relations and home life, and school environment. Alcohol abstinence was associated with higher physical wellbeing, psychological wellbeing, moods and emotions, self-perception, parent relations and home life, and school environment dimensions.

**Conclusion:**

Intervention to promote HRQoL in adolescents should consider food choices whilst encouraging physical activity, discouraging social media and deterring alcohol, and targeting boys and girls separately.

## Background

The study of quality of life has recently attracted scientific interest and gained importance in the identification of health risks and populations’ health status [1, 2]. HRQoL is a subjective construct that encompasses overall perceived health and wellbeing on sub-dimensions of physical, psychological and social functioning [3]. Adolescence is a critical period characterised by significant physiological and psychological development [4], and an important stage for the establishment of long-term health and lifestyle behaviours [5]. It is important to understand how lifestyle factors impact HRQoL during adolescence in order to develop effective health promotion strategies and interventions.

Studies that have looked at HRQoL and health outcomes during adolescence have used various measures, including the Pediatric Quality of Life Inventory [6], Kidscreen10 [7–9] and Kidscreen27 [10–12]. Few studies have employed the Kidscreen52 [13–16]. The Kidscreen52 is a self-report measure validated to assess HRQoL in both healthy and chronically ill children and adolescents aged 8–18 years [16]. The Kidscreen52 measures HRQoL on ten dimensions (physical wellbeing; psychological wellbeing; moods and emotions; self-perception; autonomy; parent relations and home life; social support and peers; school environment; social acceptance; and financial resources) (see Table 1) and has shown to have superior reliability and validity across differing settings and samples [17]. The Kidscreen52 appears to be more sensitive than shorter versions in determining associations between HRQoL and lifestyle behaviours [13, 15, 18, 19] and has been employed in the current study to enable exploration of HRQoL across the ten dimensions, enabling unpicking of different aspects of HRQoL and associations with lifestyle in adolescents in a way that is highly valid [20] and which has proven valuable in the design of HRQoL interventions [21].

**Table 1:** Interpretation of Kidscreen52 Dimensions [16]

Dimension	Definition
Physical wellbeing	Explores the level of the adolescent’s physical activity, energy, and fitness. Level of physical activity is examined with reference to the adolescent’s ability to get around the home and school, and to play or do physically demanding activities such as sports, since an adolescent’s impairment does also affect physical activity. The dimension also looks at the adolescent’s capacity for lively or energetic play. In addition, the extent to which an adolescent feels unwell and complains of poor health is examined.
Psychological wellbeing	Examines the psychological well-being of the adolescent including positive emotions and satisfaction with life. It specifically reveals the positive perceptions and emotions experienced by the individual. The questions look at how much an adolescent experiences positive feeling such as happiness, joy, and cheerfulness. It also reflects the person’s view of their satisfaction with life so far.
Moods and emotions	Covers how much the adolescent experiences depressive moods and emotions and stressful feelings. It specifically reveals feelings such as loneliness, sadness, sufficiency/insufficiency, and resignation. Furthermore, this dimension takes into account how distressing these feelings are perceived to be. This dimension shows a high score in QoL if these negative feelings are rare.
Self-perception	Explores the adolescent’s perception of self. It includes whether the appearance of the body is viewed positively or negatively. Body image is explored by questions concerning satisfaction with looks as well as with clothes and other personal accessories. The dimension examines how secure and satisfied the adolescent feels about him/herself as well as his/her appearance. This dimension is meant to reflect the value somebody assigns to him/herself and the perception of how positively others value him/her.
Autonomy	Looks at the opportunity given to an adolescent to create his/her social and leisure time. It examines the adolescent’s level of autonomy, seen as an important developmental issue for creating an individual identity. This refers to the adolescent’s freedom of choice, self-sufficiency, and independence. In particular, the extent to which the adolescent feels able to shape his/her own life as well as being able to make decisions about day-to-day activities is considered. The dimension also examines whether the adolescent feels sufficiently provided with opportunities to participate in social activities, particularly in leisure activities and pastimes.
Parent relations and home life	Examines the relationship between the parents and the atmosphere in the adolescent’s home. It explores the quality of the interaction between the adolescent and parent or carer, and the adolescent’s feelings toward parents/carers. Particular importance is attached to whether the adolescent feels loved and supported by the family, whether the atmosphere at home is comfortable or not and also if the adolescent feels treated fairly.
Social support and peers	Examines the nature of the adolescent’s relationships with other adolescents. Social relations with friends and peers are considered. The dimension explores the quality of the interaction between the adolescent and peers as well as their perceived support. The questions examine the extent to which the adolescent feels accepted and supported by friends and the adolescent’s ability to form and maintain friendships. In particular, aspects concerning communication with others are considered. It also explores the extent to which the person experiences positive group feelings and how much s/he feels part of a group and respected by peers and friends.
School environment	Explores an adolescent’s perception of his/her cognitive capacity, learning and concentration, and his/her feelings about school. It includes the adolescent’s satisfaction with his/her ability and performance at school. General feelings about school, such as whether school is an enjoyable place to be, are also considered. In addition, the dimension explores the child’s view of the relationship with his/her teachers. For example, questions include whether the adolescent gets along well with his/her teachers and whether the teachers are perceived as being interested in the student as a person.
Social acceptance	Covers the aspect of feeling rejected by peers in school. It explores both the feeling of being rejected by others as well as the feeling of anxiety toward peers. We say a student is being bullied when another student or a group of students say or do nasty and unpleasant things to him or her. It is also bullying when a student is teased repeatedly in a way he or she does not like. But it is not bullying when two students of about the same strength quarrel or fight. This definition is fairly standard and has been used over a number of years in the HBSC studies. This dimension shows a high score in QoL if these negative feelings are rare.
Financial resources	The perceived quality of the financial resources of the adolescent is assessed. The dimension explores whether the adolescent feels that s/he has enough financial resources to allow him/her to live a lifestyle which is comparable to other adolescents and provides the opportunity to do things together with peers.

Dietary studies in healthy adolescents are consistent in finding that HRQoL is associated with healthier eating [9, 12–[13, 22]–23, 24] and lower BMI [25]. Studies that have assessed HRQoL using the Kidscreen52 and food choice in non-clinical samples of adolescents have explored the Mediterranean diet (a diet characterised by high consumption of fruit, vegetables, nuts, legumes, unprocessed cereals, with low consumption of meat and dairy products) as the food choice outcome [9, 12, 26, 27]. One study that looked at diet more generally and HRQoL using the Kidscreen52 [13] found that less healthy food choices were associated with lower ratings on the dimensions of physical wellbeing and self-perception, while intake of healthier foods was associated with higher ratings on the psychological well-being, moods and emotions, self-perception and school environment dimensions.

Several studies have observed higher scores on all HRQoL dimensions in those who engage in greater physical activity [14, 28–32] implying the importance of physical activity to HRQoL. Greater physical activity has been associated with higher HRQoL on dimensions of psychological wellbeing, self-perception and school environment, and lower scores on peer and social support dimensions [28]. Physical activity has also been consistently associated with healthier food choices in adolescence [22, 33] which could contribute to its association with HRQoL.

By adolescence, between 39% [18] and 71% [19] report regularly consuming alcohol. Research using the Kidscreen10 has found no relationship between HRQoL and alcohol intake [34]. Studies that have employed the Kidscreen52 [18, 19], however, have indicated a link between alcohol intake and lower HRQoL. Those who consumed alcohol scored lower on the dimensions of psychological wellbeing [18], moods and emotions [19], self-perception [18, 19], parental relationships and home life [19] and school environment [18, 19]. This suggests that adolescent food choices and alcohol intake may be linked.

Time spent on social media has also been associated with lower HRQoL [7, 8, 35]. However, findings are not clear cut with some finding no relationship between HRQoL (assessed using the Kidscreen10) and social media use [34]. Differences in results between studies could reflect gender differences [35] and reflect the HRQoL measure employed. Research using the Kidscreen52 has produced evidence of differences across HRQoL dimensions [15, 34]. A study of Swiss adolescents (N = 895) [15] found that while less social media use was associated with higher scores on the HRQoL dimensions of ‘moods and emotions’, ‘self-perception’ and ‘parental relationships and home life’, greater social media use was associated with higher HRQoL on ‘peers and social support’. Social media appears to have both positive and negative implications for HRQoL. Another possible reason for these apparently mixed findings could be that foods, both healthy and unhealthy, feature on social media communications [36–38] and dependent on which sites are visited, could impact either positively or negatively upon food choices. This implies the potential importance of food choices in explaining links between social media use and HRQoL.

Research conducted in different countries is of the consensus that sex differences are evident in HRQoL and its dimensions, showing that adolescent girls experience lower quality of life than boys [13, 23, 39–41]. Studies that have employed the Kidscreen52 have found adolescent girls to score higher than boys on school environment and lower in physical wellbeing [12, 42]. Studies of food choice in adolescence have indicated that girls consume fruit and vegetables more often and consume meat less frequently than boys [43] and this could explain sex differences in HRQoL.

That intake of more healthy foods has been found to be associated with higher HRQoL [12, 13] as well as with greater physical activity [7, 14, 22, 28–30], lower social media use [7, 8, 15], and less alcohol use [18, 19] could imply that food choices constitute a mechanism through which healthy lifestyle practices influence HRQoL. The objective of this analysis, therefore, has been to determine the total effect of social media use, alcohol and physical activity level on HRQoL domains (measured using the Kidscreen52), over and above that explained by food choice, through an examination of direct, indirect and total effects. The aim has been to determine which lifestyle behaviours should be targeted along with diet in planning intervention to achieve positive HRQoL outcomes in adolescents. Given previous research, it is hypothesised that HRQoL will be lower in females and that higher scores on HRQoL dimensions will be associated with greater physical activity, less social media use, abstinence from alcohol, that these relationships will differ between sex and will be linked to food choice factors.

## Methods

### Design and procedure

Ethical approval was granted by the School of Education Research Ethics Committee at Queens University Belfast. Data were collected during 2016 as part of the longitudinal Wellbeing in School (WiSe) study, which explored health and wellbeing of adolescents aged 11–16 years in Northern Ireland (NI). Eighty-three secondary-level schools across NI participated. Informed consent was obtained in writing from parents and pupils. The sample comprised n = 1609 adolescents aged 13–14 years, of whom 51% (n = 821) were female. Participants completed the questionnaire electronically either on an iPad or computer, with the researcher present. One class in each year group within each school was randomly selected to participate. Detailed information on recruitment and data collection has been published previously [43, 44].

### Measures

#### Kidscreen52

Kidscreen52 [16] is a 52-item measure of HRQoL designed for use with children aged between 8 and 18 years. Kidscreen52 assesses HRQoL on ten dimensions (number of items in parentheses): physical wellbeing (5); psychological wellbeing (6); moods and emotions (7); self-perception (5); autonomy (5); parent relations and home life (6); social support and peers (6); school environment (6); social acceptance (3); and financial resources (3) (see Table 1). Responses were on five-point scales: (1) not at all, slightly, moderately, very, extremely; or (2) never, seldom, quite often, very often, always. Items were reverse-scored according to the manual so that higher scores indicated better HRQoL [17]. Dimension scores were added together and then transformed into Rasch person parameters (PP). The PPs were transformed into values with a mean of 50 and a standard deviation of 10 using the syntax provided on a CD accompanying the purchase of the KIDSCREEN manual [16–45]. The KIDSCREEN questionnaires have been shown to generate reliable results (α > 0.70) and to have good cross-national validity [21]. Reliability for the Kidscreen52 in this sample was high (α = 0.955) with dimension reliability of: physical wellbeing (0.886); psychological wellbeing (0.891); moods and emotions (0.880); self-perception (0.832) ; autonomy (0.866); parent relations and home life (0.897); social support and peers (0.860); school environment (0.888); social acceptance (0.815); and financial resources (0.908).

#### Food frequency questionnaire (FFQ)

Food choice was assessed using a 17-item food frequency questionnaire (FFQ) previously employed in the Young Persons Behaviour and Attitudes (YPBAS) Survey [46]. Items enquired as to the frequency of consumption of: sweets/ chocolate/biscuits; buns/cakes/pastries; fizzy/sugary drinks; diet drinks; crisps; chips/fried potatoes; boiled/baked potatoes; fried foods (sausage eggs, bacon); meat products; meat/meat dishes’; fish (not fried); beans/pulses; fruit; vegetables/salads (except potatoes); bread/dairy; rice/pasta; milk (to drink; on cereal; puddings) cheese/yoghurt. Responses were on a five-point scale: more than once a day; once a day; most days; once or twice a week; less often or never. The FFQ has been found to be a reliable and valid measure for assessing food choice in adolescents aged 11–15 years [47]. Reliability for the FFQ in this sample was high (α = 0.974).

The dimensional structure of responses to the 17 FFQ items was determined by means of exploratory factor analysis using a geomin (oblique) solution with chi-square testing of model fit. Because responses were on a five-point Likert scale, they were treated as ordinal. Results indicated a five-factor solution: 1 ‘Junk Food’; 2 ‘Meat’; 3 ‘Protein’; 4 ‘Fruit and Vegetables’; 5 ‘Bread/Dairy’ (see [44]). All factor loadings that were statistically significant at the 0.05 level in the exploratory factor model were then included within a five-factor solution modelled within a confirmatory framework.

#### Physical activity questionnaire for adolescents (PAQ-A)

The Physical Activity Questionnaire for Adolescents (PAQ-A) [48] is a self-administered seven-day recall questionnaire validated as a measure of physical activity in adolescents aged 14 to 20 years. It consists of nine questions for which responses are structured to discern low (1) to high (5) physical activity during the previous seven days. The first question contains an activity checklist of 22 items referring to common sports, leisure activities and games [49]. Two questions assess overall activity patterns during the week. Remaining questions assess activity during specific periods of the day, including physical education class, morning break, lunchtime, after school, evening, and weekend. The PAQ-A was scored according to the manual [48]. The average score of items was used to create a PAQ-A summary score. Reliability for the PAQ-A in this sample was high (α = 0.951).

#### Social media use

Social media use was measured by a single item which asked ‘On a normal school day, how many hours were you active on social media (i.e. from you wake to you sleep again)?’ for which response options were: (1) Less than one hour; (2) Around 1 h; (3) Around 2 h; (4) Around 3 h; (5) Around 4 h; (6) Around 5 h; (7) Around 6 h; (8) 7 h or more.

#### Alcohol abstinence

Alcohol abstinence was assessed using: ‘Have you ever had a proper alcoholic drink – a whole drink, not just a sip?’ for which response options were yes or no.

#### Sex

Biological sex was prompted by a single item: ‘Are you female or male?’ for which response options were: (1) Female; (2) Male.

### Data analysis

**Fig. 1:**
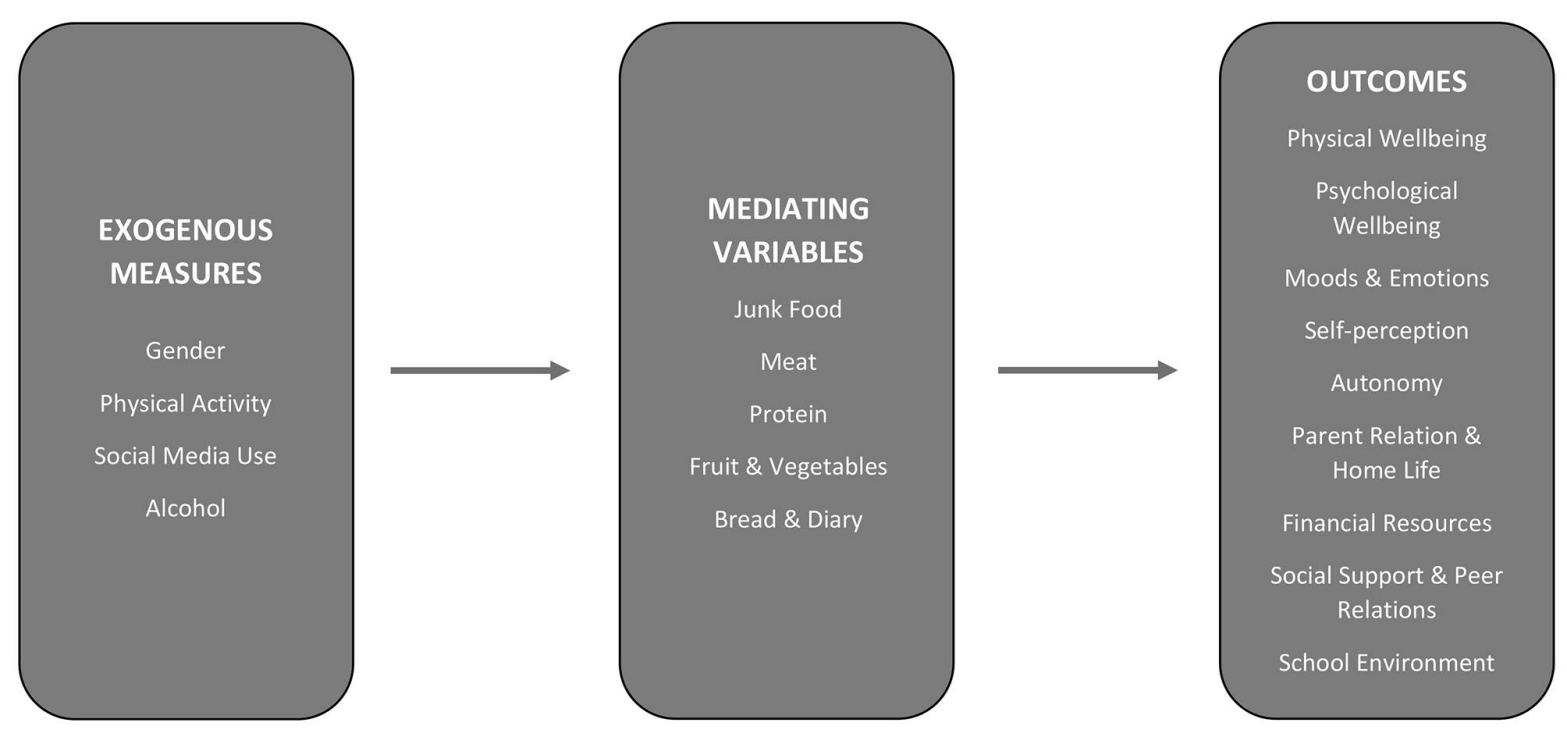
Path Analysis Model

Path analysis sought to determine the degree to which HRQoL was explained by sex, physical activity, alcohol abstinence, social media use and where mediated by food choice (see Fig. 1). The outcome variables were the 49 items comprising nine of the ten Kidscreen52 dimensions: Physical Wellbeing; Psychological Wellbeing; Moods and Emotions; Self-Perception; Autonomy; Family Relations and Home Life; Financial Resources; Social Support and Peers; School Environment. Social Acceptance was excluded from the analysis given factor analyses [21] indicating the three items constitute a separate factor distinct from the other nine and may not measure quality of life. The nine factors included in the analysis have a unidimensional structure. The nine dimensions relating to HRQoL, therefore, were evaluated with a first-order one factor model which was used to represent the relationship between the observed measures and each of the nine latent factors. Each of the nine HRQoL related factors were then taken as the outcome measures. These nine factors were then regressed onto a 5-factor model comprised of food choice factors (‘Junk Food’; ‘Meat’; ‘Protein’; ‘Fruit and Vegetables’; ‘Bread/Dairy’), which were themselves regressed on a number of explanatory measures (physical activity; social media use; alcohol abstinence) (see Fig. 1). This allowed for the direct, indirect effects and total effects. Because responses to Kidscreen52 were not equidistant and biased toward positive responses, Likert scale data were entered as ordinal variables. Given the ordinal nature of the variables, the parameter estimates are based on weighted least square mean and variance (WLSMV). This estimator is modified to accommodate missing data [50] and provides consistent estimates under general missing data assumptions [51]. There were four explanatory variables: sex (dichotomous); PAQ-A (summed total scores); alcohol abstinence (dichotomous); social media use (summed number of hours). The mediating variables were the five food choice factors. There were therefore 14 latent variables (5x food choice factors + 9x HRQoL). The nine HRQoL dimensions were the outcome variables. Analyses were conducted using Mplus VERSION 8.6 [52].

## Results

Model Indices were satisfactory: RMSEA (Root Mean Square Error of Approximation) = 0.023; CI (confidence interval) = 0.022 0.025; CFI (Comparative Fit Index) = 0.963; TLI (Tucker-Lewis Index) = 0.959; SRMR (Standardized Root Mean Square Residual) = 0.045. R Squares indicated the model explained 48% of variance in Physical Wellbeing, 15.9% of Psychological Wellbeing, 16.8% of Moods and Emotions, 28.3% of Self-Perception, 8.4% of Autonomy, 8.4% of Parental Relationships and Home Life, 6.1% of Financial Resources, 11.2% of Social Support and Peers and 15.1% of School Environment.

### Direct and indirect relationships between Sex, physical activity, social media use, alcohol abstinence, food choice factors and health-related quality of life

#### Physical wellbeing

Higher scores on physical wellbeing were directly associated with greater physical activity, greater alcohol abstinence, and more frequent intake of the bread/dairy food choice factor (Table 2).

**Table 2:** Direct effects of HRQoL on (a) exogenous measures (b) food choice factors (mediated measures) and (c) exogenous measures on food choice factors

	Physical Wellbeing (PhW) on	Psychological Wellbeing (PsyW) on	Mood and Emotion (ME) on	Self-Perception (SP) on	Autonomy on	Parent Relations/Home Life (PR/HL) on	Financial Resources (FR) on	Social Support and Peers (SSP) on	School Environment (SE) on
	**Exogenous direct effects on HRQoL**								
	**Est (SE)**	**Est (SE)**	**Est (SE)**	**Est (SE)**	**Est (SE)**	**Est (SE)**	**Est (SE)**	**Est (SE)**	**Est (SE)**
**Sex**	0.023 (0.023	0.200 (0.057)***	0.353 (0.048)***	0.881 (0.070)***	0.264 (0.057)***	0.119 (0.069)	0.082 (0.072)	-0.180 (0.060)**	-0.001 (0.065)
**Physical Activity**	0.365 (0.052)***	0.335 (0.040)***	0.160 (0.033)***	0.278 (0.041)***	0.238 (0.036)***	0.159 (0.042)***	0.206 (0.039)***	0.381 (0.033)***	0.242 (0.044)***
**Social Media Use**	-0.007 (0.004)	-0.032 (0.010)**	-0.040 (0.009)***	-0.072 (0.014)***	0.019 (0.013)	-0.035 (0.012)**	0.006 (0.014)	0.025 (0.013)*	-0.070 (0.013)***
**Alcohol Abstinence**	-0.050 (0.021)*	-0.177 (0.047)***	-0.152 (0.043)***	-0.182 (0.065)**	-0.073 (0.054)	-0.226 (0.061)***	-0.085 (0.058)	-0.052 (0.048)	-0.399 (0.060)***
	**The direct effects of food choice factors (mediating measure) on HRQoL**								
**Junk**	0.025 (0.035)	-0.131 (0.072)	-0.144 (0.072)*	-0.111 (0.089)	0.149 (0.090)	-0.104 (0.093)	-0.109 (0.096)	0.082 (0.088)	-0.160 (0.097)
**Meat**	-0.025 (0.029)	0.115 (0.051)*	1.135 (0.054)***	0.137 (0.072)*	-0.024 (0.066)	0.095 (0.074)	0.037 (0.079)	0.022 (0.062)	-0.028 (0.077)
**Protein**	-0.026 (0.037)	-0.211 (0.095)*	-0.391 (0.094)***	-0.369 (0.130)**	-0.188 (0.100)	-0.357 (0.113)*	-0.385 (0.134)**	-0.297 (0.106)**	-0.177 (0.120)
**Fruit and Veg**	0.021 (0.022)	0.036 (0.051)	0.125 (0.049)*	0.078 (0.060)	0.094 (0.057)	0.141 (0.059)*	0.192 (0.075)**	0.147 (0.058)*	0.099 (0.071)
**Bread/Diary**	0.070 (0.028)*	0.097 (0.062)	0.000 (0.057)	0.083 (0.075)	0.010 (0.060)	0.046 (0.071)	-0.021 (0.072)	0.042 (0.064)	0.100 (0.079)
	**Exogenous direct effects on food choice factors**								
	**Junk**	**Meat**	**Protein**	**Fruit and Veg**	**Bread + Diary**				
	**Est (SE)**	**Est (SE)**	**Est (SE)**	**Est (SE)**	**Est (SE)**				
**Sex**	0.202 (0.033)***	0.284 (0.054)***	0.014 (0.032)	-0.463 (0.058)***	0.005 (0.056)				
**Physical Activity**	-0.106 (0.023)***	-0.000 (0.038)	0.138 (0.023)***	0.376 (0.036)***	0.208 (0.038)***				
**Social Media Use**	0.060 (0.009)***	0.082 (0.012)***	0.012 (0.008)	-0.036 (0.011)**	0.002 (0.013)				
**Alcohol Abstinence**	0.101 (0.033)**	0.055 (0.047)	0.068 (0.026)**	0.016 (0.052)	0.041 (0.059)				

**Note:** *Est/SE ≥ 1.96, indicating statistical significance at 0.05 level; **Est/SE of ≈ > 2.57 indicating statistical significant at 0.01 level; ***Est/SE of ≈ > 3.30 indicating statistical significant at 0.001 level. Two-sided test; standard error (SE), estimate (Est)

In terms of the model, physical activity was indirectly related to physical wellbeing via more frequent choice of the bread/dairy food choice factor (est = 0.015, se = 0.006, t = 2.349, p = 0.019).

#### Psychological wellbeing

Higher scores on psychological wellbeing were directly associated with being male, greater physical activity, less use of social media, greater alcohol abstinence, more frequent intake of meat and less frequent intake of the protein factor (Table 2).

The relationship between higher psychological wellbeing and being male was mediated by more frequent intake of meat (est = 0.033, se = 0.016, est/se = 2.055, p = 0.040). More frequent intake of meat was also a mediating factor between less use of social media and higher psychological wellbeing (est = 0.009, se = 0.004, est/se = 2.106, p = 0.035). The mediating effect of a less frequent intake of protein reduced the strength of the association between physical activity and psychological wellbeing (est=-0.029, se = 0.014, est/se=-2.149, p = 0.032).

#### Moods and emotion

Higher moods and emotion scores were directly associated with being male, greater physical activity, greater alcohol abstinence, less social media, less frequent intake of the junk food factor, less frequent protein, more frequent meat intake and more frequent intake of fruit and vegetables (Table 2).

The relationship between higher moods and emotion and being male was mediated by less frequent intake of junk food (est=-0.029, se = 0.015, est/se=-1.998, p = 0.046), less frequent intake of fruit and vegetables (est=-0.058, se = 0.024, est/se=-2.411, p = 0.016) and more frequent intake of meat (est = 0.038, se = 0.016, est/se = 2.419, p = 0.016).

Greater physical activity was associated with a higher moods and emotion and this was mediated by more frequent intake of fruit and vegetables (est = 0.047, se = 0.018, est/se = 2.635, p = 0.008) and less frequent intake of the protein factor (est=-0.054, se = 0.015, est/se=-3.676, p = 0.000).

Less use of social media and higher moods and emotion was mediated by more frequent intake of the meat factor (est = 0.011, se = 0.004, est/se = 2.594, p = 0.009) and less frequent intake of fruit and vegetables (est=-0.004, se = 0.002, est/se=-2.092, p = 0.036).

The direct association between alcohol abstinence (greater) and higher moods and emotion was mediated by less frequent intake of the protein factor (est=-0.027; se = 0.012; est/se=-2.223; p = 0.026).

#### Self-perception

Higher self-perception scores were directly associated with being female, greater physical activity, less social media, greater alcohol abstinence, and less frequent intake of the protein factor (Table 2).

The association between higher self-perception and greater physical activity was mediated by less frequent consumption of protein (est=-0.051, se = 0.020, est/se=-2.599, p = 0.009) and more frequent intake of meat (est=-0.025, se = 0.013, est/se=-1.974, p = 0.048).

#### Autonomy

Higher scores on Autonomy were directly associated with being female and greater physical activity (Table 2). The autonomy scores were neither directly nor indirectly associated with any of the food choice factors.

#### Parental relations and home life

Higher scores on parental relations and home life were directly associated with being male, greater physical activity, less social media use, greater alcohol abstinence, less frequent consumption of protein and more frequent intake of fruit and vegetables (Table 2).

The higher scores on parental relations and home life in males was mediated by less frequent consumption of fruit and vegetables (est=-0.065, se = 0.029, est/se=-2.264, p = 0.024).

The association between higher scores on parental relations and home life and greater physical activity was mediated by less frequent intake of the protein factor (est=-0.049, se = 0.018, est/se=-2.799, p = 0.005) and by more frequent intake of the fruit and vegetables factor (est = 0.053, se = 0.022, est/se = 2.409, p = 0.016).

Greater alcohol abstinence and higher parental relations and home life was mediated by less frequent intake of protein (est=-0.024, se = 0.011, est/se=-2.309, p = 0.021).

#### Financial resources

Higher scores on financial resources were directly associated with greater physical activity, less frequent consumption of protein and more frequent intake of fruit and vegetables (Table 2).

Although there was no direct association between sex and financial resources, those more frequently consuming fruit and vegetables were more likely to be female and to have higher financial resources (est=-0.089; se = 0.035; est/se=-2.558; p = 0.011).

Higher financial resources and greater physical activity was mediated by less frequent intake of protein (est=-0.053, se = 0.019, est/se=-2.747, p = 0.006) and more frequent intake of fruit and vegetables (est = 0.072, se = 0.029, est/se = 2.516, p = 0.012).

The association between social media use and financial resources was mediated by less frequent intake of the fruit and vegetables (est=-0.007, se = 0.003, est/se=-1.982, p = 0.047).

Although there was no direct association between alcohol abstinence and financial resources, lower alcohol abstinence was associated with less frequent intake of protein and a lower financial resources score (est=-0.026, se = 0.013, est/se=-2.024, p = 0.043).

#### Social support and peers

Higher scores on social support and peers were directly associated with being female, greater physical activity, greater social media use, more frequent consumption of protein and more frequent intake of fruit and vegetables (Table 2).

The association between being female on social support and peers was mediated by less frequent intake of fruit and vegetables (est=-0.068, se = 0.028, est/se=-2.402, p = 0.016).

The association between higher social support and peers and greater physical activity was mediated by (a) less frequent intake of protein (est=-0.041, se = 0.014, est/se=-2.948, p = 0.003) and (b) more frequent intake of fruit and vegetables (est = 0.055, se = 0.022, est/se = 2.542, p = 0.011).

Higher social support and peers scores and the association with greater use of social media was mediated by less frequent intake of the fruit and vegetables (est=-0.005, se = 0.003, est/se=-1.977, p = 0.048).

While there was no direct association between protein on social support and peers, the association between alcohol abstinence and higher social support and peers was mediated by less frequent intake of protein (est=-0.020, se = 0.010, est/se=-2.084, p = 0.037).

#### School environment

Higher scores on school environment were directly associated with greater physical activity, less social media use and greater alcohol abstinence (Table 2). School environment was unrelated to sex or to food choice factors. There were no indirect relationships between the food choice factors and school environment.

### Food choice factors, sex, physical activity, Social Media Use and Alcohol abstinence (partial regression coefficients)

More frequent intake of the Junk food factor was directly associated with being male, less physical activity, greater social media use and lower alcohol abstinence. Frequent intake of the Meat factor was directly associated with being male and greater social media use. Frequent intake of the Protein factor was directly associated with greater physical activity and lower alcohol abstinence. Frequent intake of the Fruit and Vegetable factor was directly associated with being female, greater physical activity and less social media use. More frequent intake of the Bread/Dairy factor was directly associated with greater physical activity.

## Discussion

This study sought to determine the degree to which relationships between HRQoL dimensions and physical activity, social media use and abstinence from alcohol, are mediated by food choice factors. As predicted and consistent with previous research [13, 23, 39–41] males had higher HRQoL on the psychological wellbeing, moods and emotions and parental relations and home life dimensions. Previous studies that have assessed HRQoL using the Kidscreen52 have also found that males scored higher on parental relations and home life [13, 39], school environment [39], physical wellbeing [13, 39], self-perception [13] and autonomy [13, 39]. This analysis indicated that females had higher HRQoL than males on the dimensions of self-perception, autonomy and, social support and peers. Previous research has consistently pointed to sex differences in adolescent dietary habits [43]. Sex differences between Kidscreen52 HRQoL dimensions, therefore, could be related to mediating food choice factors. In support of this notion, males who consumed fruit and vegetables more frequently and junk food less frequently and had better HRQoL on the moods and emotions dimension. This agrees with previous research indicating that less healthy eating is underpinned by negative emotions [53, 54]. Previous research has also indicated that adolescent males consume more meat than females [44, 53] and this analysis indicates that males who consumed meat more frequently had higher HRQoL on the psychological wellbeing and moods and emotions dimensions. Intervention to enhance HRQoL in males should therefore seek to deter consumption of junk food and encourage greater intake of meat, fruit and vegetables. That the relationship between being male and higher parental relations and home life was mediated by less frequent consumption of fruit and vegetables could reflect parental approach to food provision [55] and will require further study. Although no direct relationship, that females who consumed fruit and vegetables more frequently had higher HRQoL on financial resources and social support and peers, implies that females could also benefit from promotion of fruit and vegetable intake.

As predicted and consistent with previous research [14, 29] greater physical activity was associated with higher HRQoL on all nine dimensions. This corroborates results of other studies in adolescence indicating that greater physical activity is associated with better HRQoL [8, 14, 28, 29, 22]. Together this highlights the importance of physical activity to adolescents HRQoL and the imperative to promote physical activity. That adolescents who eat more fruit and vegetables are more likely to engage in physical activity is well documented [56–61]. Previous research has also recorded high protein intake in physically active adolescents [62]. It was therefore not surprising to find that associations between greater physical activity and better HRQoL on the moods and emotions, parental relations and home life, financial resources and social support and peers dimensions were mediated by more frequent fruit and vegetable and less frequent protein intake. The association between greater physical activity and higher self-perception was also mediated by less frequent protein intake.

These findings indicate a link between better HRQoL and physical activity especially where there is less frequent protein intake. That the relationship between psychological wellbeing and greater physical activity was weakened by more frequent intake of the protein factor, could imply that when physical activity is coupled with high protein it is less advantageous to psychological wellbeing. These findings should be interpreted cautiously given protein intake can be difficult to assess accurately [63]. Those who engaged in greater physical activity and had better physical wellbeing reported more frequent intake of bread and dairy foods. That the model for physical wellbeing was the strongest gives weight to these findings.

Abstinence from alcohol was associated with higher HRQoL on six out of the nine dimensions (physical wellbeing; psychological wellbeing; moods and emotions; self-perception; parental relations and home life and school environment). This finding was as expected and consistent with other studies using the Kidscreen52 which also found that those with higher alcohol intake scored lower on psychological wellbeing [20], moods and emotions [21], self-perception [20, 21], parental relations and home life [21] and school environment [20, 21]. Abstinence from alcohol was associated with higher moods and emotions, parental relations and home life and lower financial resources, all of which were mediated by less frequent protein intake. Although no direct relationship, lower alcohol abstinence was indirectly related to lower financial resources and greater alcohol abstinence was indirectly related to higher social support and peers, both of which were also mediated by less frequent protein intake. This implies that intervention to enhance HRQoL should seek to deter alcohol and increase protein intake in those in less affluent circumstances. Again, care needs to be taken in interpreting associations with protein given inaccuracies in assessing protein intake accurately [50].

Greater social media use was associated with lower HRQoL on six dimensions (psychological wellbeing, moods and emotions, parental relations and home life, social support and peers, school environment). This concurs with findings of previous studies linking greater screen time to lower HRQoL [7, 8], but contrary to previous research [15], indicating that higher HRQoL on dimensions of moods and emotions, self-perception and parental relations and home life is associated with *less* social media use. That greater social media use was associated with higher social support and peers corroborates Foerster and Roosli’s [15] result. Social media use appears to be both detrimental and beneficial to HRQoL depending upon the dimension. Inconsistency in the literature could reflect cultural differences between samples and warrants further research in diverse populations.

The only previous survey that appears to have investigated links between diet and HRQoL using the Kidscreen52 sampled 10-17-year-olds (N = 669) in Portugal [13] and concluded that intake of less healthy food was associated with lower HRQoL. The adolescents who took part in our study and who reported greater use of social media, also consumed junk food and meat frequently. Those who used social media less, reported consuming fruit and vegetables more frequently. This suggests that to discourage intake of junk food and encourage intake of fruit and vegetables we need to look to social media and intervene so that adolescents become more wary of content.

There were also direct relationships between the food choice patterns and HRQoL domains. Adolescents who ate bread more frequently reported better psychological wellbeing and those who consumed junk food less frequently were higher on the moods and emotions dimension. Previous research that investigated links between diet and HRQoL using the Kidscreen52 [13] also identified associations between food consumed and higher scores on psychological wellbeing and moods and emotions dimensions. More frequent fruit and vegetable intake was associated with higher moods and emotions, parental relations and home life, financial resources and social support and peers. The relationship between more frequent fruit and vegetable intake and certain HRQoL domains is in keeping with previous research linking healthy eating to better psychological wellbeing [43], greater family affluence [44] and peer influence [53]. The finding that those who consumed protein less frequently had better HRQoL on the physical wellbeing and psychological wellbeing, moods and emotions, parental relations and home life, financial resources and social support and peer dimensions is novel and will require further research.

The cross-sectional survey design and correlational nature of this analysis limits the degree to which conclusions can be drawn on the causative relationships between HRQoL, lifestyle and food choice factors. Although not possible to establish cause and effect this analysis, these findings imply that to enhance HRQoL, adolescents should be encouraged to increase fruit and vegetable intake and restrict protein-rich foods. That diet was the only lifestyle factor associated with financial resources implies it could be an important mediating factor in HRQoL in disadvantaged youth. Higher psychological wellbeing and moods and emotions were associated with less social media use and these relationships were mediated by more frequent meat intake. The possibility that meat eating is a surrogate for better HRQoL on psychological dimensions requires further study. Another potential limitation of this study lies in the degree of inaccuracy and bias inherent in self-assessed diet [63]. Together, these results emphasise the importance of diet to HRQoL. A strength of this analysis is that it has considered HRQoL as comprised of separate dimensions (rather than as a unified factor) using Kidscreen52 and has sought to establish how the various dimensions relate to lifestyle behaviour of adolescents. Kidscreen52 is considered a reliable measure of HRQoL and has been validated in a large sample (N = 22,827) aged 8–18 years across 13 EU countries [21]. Studies that have looked at HRQoL and health outcomes in adolescents, however, are difficult to compare given only a few have employed the Kidscreen52 and compared lifestyle between HRQoL dimensions. This study is also novel that it has explored the potential mediating role of food choice associations between lifestyle and HRQoL dimensions.

These findings add to a growing body of literature indicating that greater physical activity, greater alcohol abstinence and less social media use are associated with better HRQoL in adolescents, suggesting that these behaviours could be targeted together to enhance HRQoL. That females scored lower than males on psychological wellbeing, moods and emotions, and parental relations and home life implies an imperative to target girls specifically on these dimensions. The results also support the notion that food choices act as a surrogate and/or serve as a mechanism though which physical activity, alcohol abstinence and social media use relate to HRQoL. These findings imply that food choices need to be considered when promoting HRQoL in adolescents.

## Data Availability

The datasets used and analysed during the current study is available from the corresponding author on reasonable request.
